# A Case Report of Esophageal Perforation: Complications of Orogastric Tube Placement

**DOI:** 10.7759/cureus.33535

**Published:** 2023-01-09

**Authors:** Yogesh Subedi, Biplov Adhikari, Ashik Pokharel, Kalyan Poudel, Sajja Sharma

**Affiliations:** 1 Internal Medicine, MedStar Union Memorial Hospital, Baltimore, USA; 2 Internal medicine, MedStar Union Memorial Hospital, Baltimore, USA; 3 Radiology, Medstar Health, Baltimore, USA; 4 Internal Medicine, Nepalese Army Institute of Health Sciences, Kathmandu, NPL

**Keywords:** complication, iatrogenic injury, pneumomediastinum, orogastric tube, esophageal perforation

## Abstract

Orogastric tube (OGT) insertion is a routine procedure in medical care. It is often inserted in patients after endotracheal intubation. OGT insertion is often a blind procedure. Misplacement of the tube can cause a variety of complications and can sometimes be life-threatening. We present the case of a 71-year-old male patient who experienced a rare proximal esophageal perforation as a complication of blind insertion of the OGT; he required OGT insertion after receiving endotracheal intubation for hypoxic respiratory failure secondary to COVID-19 infection. The esophageal perforation was revealed on a post-procedural roentgenogram and confirmed by a subsequent computed tomography of the chest. Given the small size of the perforation and the absence of clinical instability, conservative management was pursued leading to improvement of the mediastinitis. Although the complications of OGT insertion are uncommon, their consequences can be potentially serious and require a high degree of suspicion.

## Introduction

Esophageal perforation is a life-threatening condition that is associated with high morbidity and mortality [1]. It can be iatrogenic or spontaneous and can occur in various parts of the esophagus. Iatrogenic perforation can cause due to instrumentation for diagnostic or therapeutic purposes such as endoscopy, stent placement, hemostasis, foreign body removal, and pneumatic dilatation, while spontaneous perforation can occur due to a sudden rise in intra-esophageal pressure along with negative intra-abdominal pressure [2]. Diagnosis of perforation may often be delayed [2], which can be catastrophic. We present a case of esophageal perforation, diagnosed through radiographic imaging, caused by orogastric tube (OGT) placement. We previously presented this article as an abstract poster at the 2022 American College of Gastroenterology meeting on October 23, 2022.

## Case presentation

**Figure 1:**
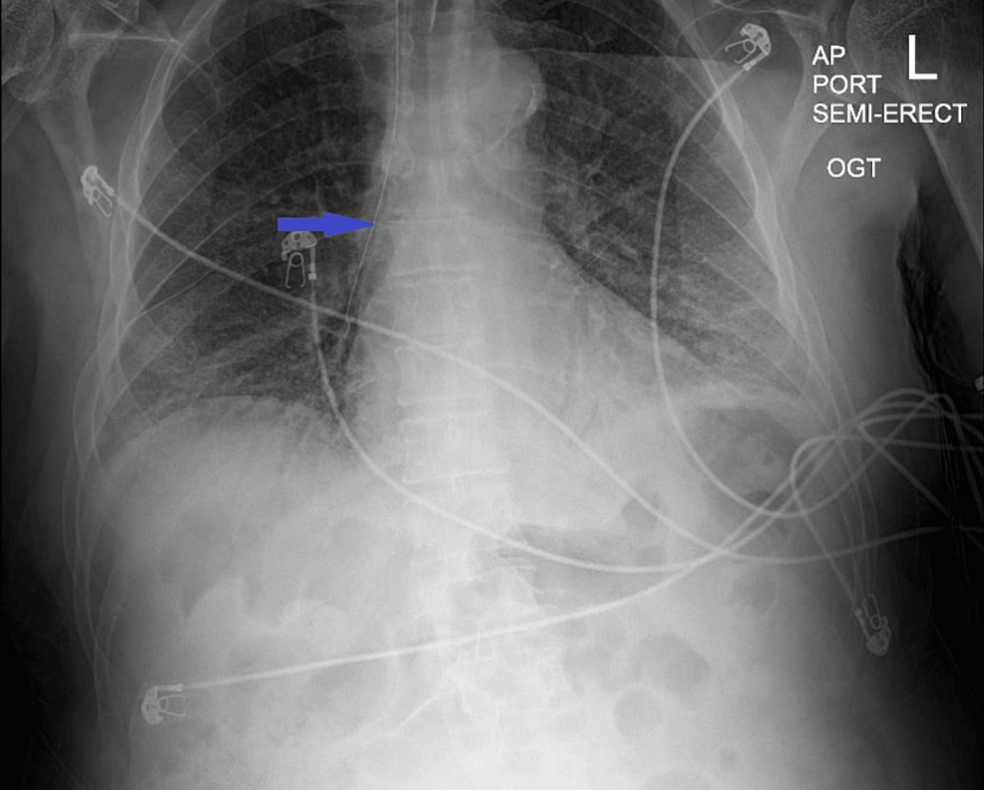
Antero-posterior X-ray showing pneumomediastinum with an orogastric tube in-situ.

**Figure 2:**
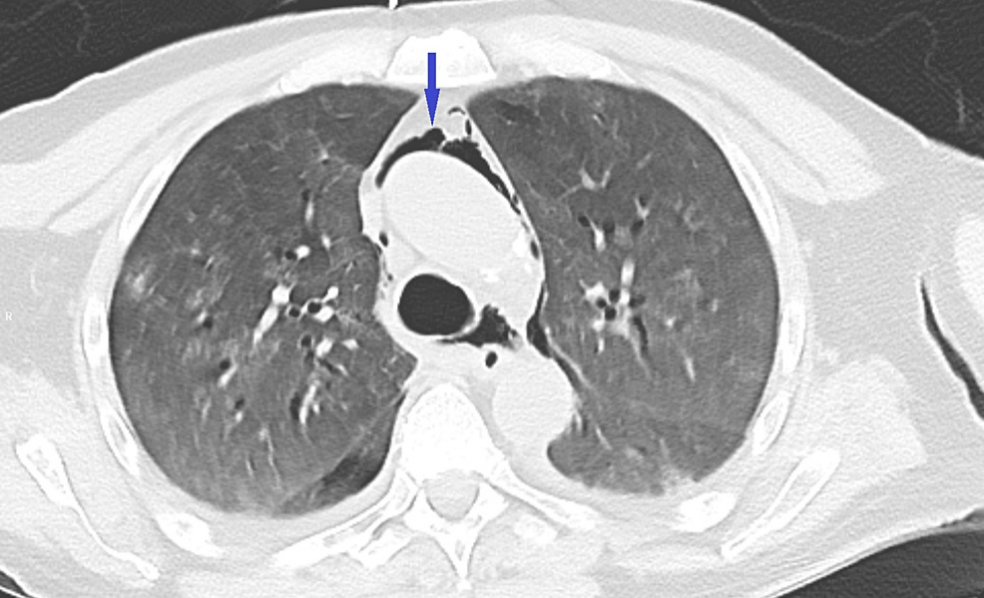
CT of the chest (axial view) showing pneumomediastinum.

We present the case of a 71-year-old male patient who had no known medical illnesses and presented with a two-week history of dyspnea, fever, chills, cough, and myalgia. His initial vital signs were notable for tachycardia and tachypnea; he was hypoxic to 66% on room air. He was diagnosed with COVID-19 pneumonia; subsequently, he was placed on a high-flow nasal cannula (HFNC), started on dexamethasone, and admitted to the intensive care unit (ICU) for the management of acute hypoxic respiratory failure secondary to COVID-19 pneumonia. He continued to remain hypoxic despite being on maximal HFNC and was intubated on the seventh day of admission. Post-intubation OG placement was unsuccessful on the first attempt due to resistance. On the second attempt, the nurse was able to advance partially. However, a chest X-ray showed OGT in the mediastinum, and thus, the tube was removed (Figure 1). CT of the neck and chest without contrast was performed and revealed pneumomediastinum with possible mid-thoracic esophageal perforation (Figure 2). The patient was started on broad-spectrum antibiotics. General surgery and thoracic surgery were consulted for esophageal perforation. Given the patient’s need for mechanical ventilation, thoracic surgery deemed him unfit to tolerate thoracotomy, and the endoscopic procedure was not available in the hospital. As it was a small iatrogenic perforation, the patient was managed conservatively. Although his pneumomediastinum was gradually resolving, his hospital stay was complicated by episodes of hypotension, requiring vasopressor intervention. He also developed metabolic acidosis in the setting of acute renal failure and was started on continuous renal replacement therapy (CRRT). The patient’s code status was changed by his family to Do Not Resuscitate (DNR) due to his deteriorating condition. Eventually, he had a pulseless electrical activity (PEA) arrest and succumbed to his illness. 

## Discussion

Nasogastric and orogastric tube (NGT/OGT) placement is a common procedure performed routinely in clinical anesthesiology and the ICU. It is commonly used for gastric decompression, medicine administration, and enteral feeding [3]. The most common conventional method of OGT placement is through blind insertion, which is associated with low first-pass success with frequent complications [4]. An anteroposterior chest radiograph is a gold standard for the confirmation of tube placement. The tube must follow the course of the esophagus and avoid contours of the trachea and bronchi, clearly bisect the carina or bronchi, must cross the diaphragm in the midline and curve to the left while entering the body of the stomach below the diaphragm [5].

Although considered a safe procedure, complications may arise due to trauma or misplacement toward the endotracheal and intracranial planes. Complications can include nose bleeds, sinusitis, tracheobronchial perforations, pulmonary aspiration, pneumothorax, tube knotting, esophageal perforation, and even intracranial insertion [6]. If there is misplacement in the upper gastrointestinal tract, the tube will crook in the oropharynx or esophagus. In contrast, traumatic submucosal placement may result in partial esophageal perforation or gastric perforation that can be complete [7]. 

The clinical presentation of esophageal perforation depends on several factors, including perforation etiology, site, severity of contamination, damage to adjacent mediastinal structures, and elapsed time from perforation to treatment. Cervical esophageal perforation may present as neck pain, dyspnea, dysphagia, dysphonia, and crepitus or tenderness on palpation of the neck. Thoracic esophageal perforation can cause chest pain, dyspnea, tachypnea, nausea, and vomiting, as well as crepitus on chest wall palpation, dullness on chest percussion, decreased fremitus, and mediastinal crackling on auscultation. In the case of abdominal esophageal perforation, a patient can have epigastric pain radiating to the shoulder, along with nausea, vomiting, and signs of peritonitis, if the peritoneum is contaminated [8].

Mortality risk associated with esophageal perforation is as high as 65% due to difficulty in approaching the esophagus, lack of a strong serosa, extraordinary blood flow within the organ, and proximity to vital organs [9,10]. About 70% of esophageal perforations are iatrogenic secondary to instrumentation, with spontaneous perforations, foreign bodies, and trauma accounting for 15%, 8%, and 5% of the cases, respectively [11]. In a study of 2564 patients in England, spontaneous esophageal perforation accounted for 81.9% of the cases and iatrogenic perforation for 5.9%. Patients with esophageal perforation had 30- and 90-day mortality rates of 30% and 38.8%, respectively, and a significant portion of these patients received supportive treatment [12]. The majority of perforations happen in the distal intrathoracic or abdominal esophagus, and a prompt diagnosis requires a high index of clinical suspicion, as well as confirmation by laboratory and radiographic imaging [13,14]. As mentioned above, in our patient’s case, the first attempt at OGT placement was unsuccessful, and on the second attempt, it was advanced partially but arose the suspicion of OGT misplacement. With our patient, his advanced age leading to esophageal laxity may also have contributed to an increased risk of perforation. The mechanism of esophageal perforation in our patient was most likely mechanical trauma in the setting of forceful insertion of the OGT. Interestingly, the perforation, in this case, was in the esophagus’ proximal portion, which is less commonly reported in the literature. 

Given the size of the perforation in our patient, no active surgical intervention was performed, and the patient was stabilized medically, exhibiting gradual improvement of the pneumomediastinum. The management of esophageal perforation requires advanced endoscopic techniques that may not be available in community hospitals, as was the case with our patient. When there are no clinical symptoms of sepsis, conservative care has shown to be effective in a small subset of patients, although surgery is still the preferred course of action in the case of clinical instability [15]. In our patient, although his mediastinitis improved with conservative management, his COVID-19 infection led to hypoxia and subsequent multiorgan dysfunction, which was the ultimate cause of his death.

## Conclusions

We described a rare case of proximal esophageal perforation in an intubated patient, which was managed conservatively. Complications of OGT insertion are uncommon; however, the consequences can be potentially serious. The anatomy of the upper gastrointestinal system should be understood by the provider involved in the care; the care provider must be careful not to apply excessive force even if counter-traction is not felt. Esophageal perforations can be managed conservatively when the perforation is small without signs of clinical instability; thoracic surgery or advanced endoscopic repair is required in the case of clinical instability.
